# Weight stability in adults with obesity initiating medical marijuana treatment for other medical conditions

**DOI:** 10.1186/s42238-022-00157-6

**Published:** 2022-08-27

**Authors:** Michelle R. Lent, Meghan Visek, Paulina Syracuse, Karen L. Dugosh, David S. Festinger

**Affiliations:** 1 School of Professional and Applied Psychology, Philadelphia College of Osteopathic Medicine, 4190 City Avenue, Philadelphia, PA 19131 USA; 2 Research & Evaluation Group, Public Health Management Corporation, 1500 Market Street, Philadelphia, PA 19102 USA

**Keywords:** Obesity, Body weight, Medical marijuana, Clinical care

## Abstract

Few studies have evaluated weight change in patients who initiate medical marijuana treatment to address diagnosed health concerns. The objective of this study was to examine whether patients initiating medical marijuana use for a qualifying health condition experienced changes in health and biopsychosocial functioning over time, including weight gain or loss. Specifically, this observational, longitudinal study evaluated changes in the body mass index (BMI) of adults with co-morbid obesity (body mass index [BMI] ≥ 30 kg/m^2^) and severe obesity (BMI ≥ 40 kg/m^2^) who were starting medical marijuana treatment for any of the 23 qualifying medical conditions at one of three dispensaries in Pennsylvania. Height and weight measurements were collected at baseline (prior to medical marijuana use) and then 90 days (± 14 days) later. Participants included in analyses (*n* = 52, *M* = 55.0 ± 13.6 years, 59.6% female) had a mean baseline BMI of 36.2 ± 5.4 kg/m^2^ and the majority sought medical marijuana for chronic pain (73.1%). No significant change in BMI was observed from baseline to month three (*p* > 0.05) in the sample. Additionally, no significant change in BMI was observed in the subset of patients with severe obesity (*n* = 12, *p* > 0.05). Our findings are limited by low follow-up rates and convenience sampling methodology but may help to mitigate weight gain concerns in the context of medical marijuana use.

**Supplementary Information:**

The online version contains supplementary material available at 10.1186/s42238-022-00157-6.

Access to medical marijuana continues to expand in the USA (Han et al. 6), with 36 states and 4 territories currently allowing its use (National Conference of State Legislators 8). However, little is known about the relationship between the use of medical marijuana and weight change in patients with obesity initiating medical marijuana therapy for qualifying conditions that frequently co-occur with obesity, such as chronic pain, anxiety disorders, and neuropathies (Okifuji and Hare 10; Scott et al. 14; Central obesity is associated with neuropathy in the severely obese 3). On one hand, medical marijuana users may experience improvements in symptoms, resulting in greater mobility and caloric expenditure (Sabia et al. 12), and in turn, potential reductions in body weight. Conversely, and in accordance with the “munchies” hypothesis (Beulaygue and French 2), marijuana use may stimulate appetite in some patients (Gerich et al. 5; Waissengrin et al. 15)or be associated with reductions in initiative (aka, “amotivation”) (Sabia et al. 12), contributing to potential increases in body weight. Overall, the relationship between Body Mass Index (BMI) and marijuana is not well-understood (Sabia et al. 12; Beulaygue and French 2; Rodondi et al. 11; Sansone and Sansone 13). While some studies suggest lower BMI among users relative to non-users (Beulaygue and French 2; Smit and Crespo 15), higher caloric intake in users is also reported (Smit and Crespo 15).

As the rate of obesity remains high (Ogden et al. 9), and the prevalence of medical marijuana therapy continues to increase (Han et al. 6), it is important for providers to better understand the relationship between medical marijuana use and weight change as it would help to inform clinical obesity care (Diggins and Heinberg 4). To date, several studies of weight-loss surgery patients suggest no differences in weight outcomes between marijuana users and non-users postoperatively (Shockcor et al. 2021; Bauer et al. 1; Jung et al. 7); however, these studies did not distinguish between recreational and medicinal products. Given the oversight and regulations associated with medical marijuana dispensing (including patient qualifications, mode of delivery, dosing, and formulation), studies of weight change in recreational marijuana users may not be generalizable to medical marijuana users.

This paper used data from a larger ongoing observational study of the relationship between medical marijuana treatment initiation and biopsychosocial functioning over time. The primary objective of this sub-study was to examine three-month post-initiation weight change in a sample of patients with obesity (BMI ≥ 30.0 kg/m^2^) who began medical marijuana therapy for one of the 23 qualifying conditions in Pennsylvania (Supplementary Table 1). As a secondary objective, weight change in the subset of patients with severe obesity (BMI ≥ 40.0 kg/m^2^) was also evaluated.

## Method

The Institutional Review Board of the Philadelphia College of Osteopathic Medicine (#H17-060) provided oversight of this prospective, observational study. Participants were recruited from three medical marijuana dispensaries located in the greater Harrisburg, Pennsylvania area as a part of a larger longitudinal study of the impact of medical marijuana therapy on quality of life. Eligible participants were at least 18 years of age, had a state-issued medical marijuana card, and were naïve to medical marijuana therapy (a history of recreational use was allowed). Potential participants were identified by the dispensary pharmacists during new patient consultations. Interested patients were then referred to onsite study staff. As part of the informed consent process, potential participants were informed that the study objective was to evaluate how medical marijuana affects people’s health and functioning in different areas of their lives. After participants provided informed consent, staff collected their demographic information along with their height (inches) and weight (lbs.) (SECA 813, SECA Corp., Chino, CA) with no shoes and light clothing. Participants returned in 90 days (± 14 days) to complete a follow-up assessment that included weight measurement. Participants received $25 for each study visit and a discount on their medical marijuana purchases. To be included in the analytic sample, individuals had to meet the established definition of obesity (BMI ≥ 30.0 kg/m^2^) and have completed both baseline and 3-month assessments. The datasets analyzed are available in the DRYAD repository, https://datadryad.org/stash/share/vSsOklmIZBZOivCTILHPS7eAhlVXgw_lTvO_z3ACm64.

We calculated descriptive statistics (e.g., means and standard deviations, frequencies and percentages) on a number of baseline demographic and status variables to characterize the sample. We performed paired *t*-tests to examine changes in BMI from baseline to the 3-month follow-up for the overall sample of patients with obesity and for a subsample of patients who were identified as living with severe obesity. To understand the impact of attrition on the generalizability our findings, we conducted a series of independent t-tests to identify systematic differences among participants who did not attend the 3-month follow-up. Cohen’s *d* was calculated as a measure of effect size. Data were analyzed using SPSS version 24.0 (IBM). It should be noted that data from the baseline and month three assessment interviews were used in the current analyses as it was our goal to examine the early association between medical marijuana treatment initiation and body weight.

## Results

At the time of this report, 290 patients were approached for participation in the larger trial and 265 enrolled (91.4%). Within this larger sample, 115 participants were identified as having a BMI ≥ 30 kg/m^2^ at baseline, with 33 (28.7%) meeting the criterion for severe obesity (BMI ≥ 40.0 kg/m^2^). The majority of these 115 participants were recommended medical marijuana to treat chronic pain (65.2%) and/or anxiety (50.4%). A total of 52 participants (*M* = 55.0 ± 13.6 years, 59.6% female, 96.2% White, 73.1% chronic pain) attended the 3-month follow-up assessment (45.2% follow-up rate) and were included in analyses. All of these individuals reported that they were still engaged in medical marijuana therapy. Participants who completed the three-month follow-up assessment were significantly older (*t*(113) = − 2.77, *p* = 0.007) than those who did not complete three-month height and weight assessments (*M* = 47.4 ± 15.4 years). The two groups did not differ on any of the other of the baseline variables examined (all *p*s > 0.05).

At the time of study entry, participants reported the most common purchases at the dispensaries to be tinctures (61.5%), topicals (38.5%), capsules (40.4%), disposable pens (17.3%), and cartridges (76.8%) (multiple product purchases possible). All products contained THC or a combination of THC and CBD. At 3 months, all participants included in analyses endorsed continued use of medical marijuana.

No significant differences in BMI were found from baseline (*M* = 36.2 ± 5.4 kg/m^2^) to 3 months (*M* = 36.6 ± 5.9 kg/m^2^) for the 52 participants with obesity that completed both assessments to the 3-month follow-up assessment, *t*[51] = − 0.89, *p* > 0.05, *d* = − 0.07. Similar null results were observed for the subset of patients with severe obesity (*n =* 12). BMI values were not significantly different at baseline (*M* = 44.4 ± 4.0 kg/m^2^) and 3-month follow-up (*M* = 44.0 ± 4.8 kg/m^2^) for this subgroup of participants, *t*(11) = 1.22, *p* > 0.05, *d* = 0.10.

## Discussion

The current study found that patients with obesity initiating medical marijuana treatment for any of the qualifying conditions in Pennsylvania, most commonly chronic pain and anxiety, did not experience significant weight changes over the first 3 months of medical marijuana use. These findings provide preliminary evidence to help alleviate concerns from patients with obesity, or their recommending physicians, that medical marijuana may adversely impact weight. Further, behavioral weight management patients presenting with conditions that frequently co-occur with obesity, such as chronic pain (Okifuji and Hare 10), may be able to continue medical marijuana during weight-loss treatment without concerns for medical marijuana-related weight gain. Given our similarly null findings in the small subset of patients with severe obesity as well, weight loss surgery providers could consider restarting medical marijuana in postoperative patients when medically indicated. However, our findings are notably limited by our small samples and underpowered analyses and therefore represent an initial step in our understanding of weight change in the context of medical marijuana therapy.

Our study findings are also limited by the observational design, predominantly White sample, convenience sampling, and the low follow-up rate at 3 months (45.2%) due to face-to-face restrictions placed on dispensaries at times during the pandemic. Specifically, patients were not permitted inside the dispensaries for the majority of the duration of this study due to COVID-19, and therefore, follow-up weights were not obtained from patients who did not feel comfortable attending outdoor, curbside study visits due to COVID-19 concerns. Additionally, participants may have been weighed at different times of the day at baseline and 3 months, which could impact weight outcomes. Additionally, it is possible that informing participants of the study’s objective (i.e., to examine changes in various health outcomes over time) could have created a demand characteristic resulting in participants being more mindful of their caloric intake or expenditure during the study duration. Studies with more rigorous experimental designs that incorporate larger and more diverse samples, measures of dietary intake and physical activity, and longer follow-up intervals are needed to further clarify the relationship between medical marijuana therapy and weight change in patients with obesity. As the body of marijuana-focused research continues to expand, differentiating between studies of recreational and medicinal users could be of benefit given that medicinal users may have different reasons for, and expectations of, marijuana use. Furthermore, medicinal users are using marijuana products with known profiles and are under the care of providers.

## Supplementary Information

**Additional file 1: Table 1.** List of Qualifying Conditions for Medical Marijuana in Pennsylvania*.

## Data Availability

The dataset analyzed during the current study is available in the DRYAD repository, https://datadryad.org/stash/share/vSsOklmIZBZOivCTILHPS7eAhlVXgw_lTvO_z3ACm64.
