# Supplementary material for: Weight stability in adults with obesity initiating medical marijuana treatment for other medical conditions
Source: J Cannabis Res. 2022 Aug 27;4:48. doi: 10.1186/s42238-022-00157-6 (PMC9418648; doi:10.1186/s42238-022-00157-6)
Supplement: Supplementary file 1 — Additional file 1: Table 1. List of Qualifying Conditions for Medical Marijuana in Pennsylvania*. [file 42238_2022_157_MOESM1_ESM.docx]

Table 1. List of Qualifying Conditions for Medical Marijuana in Pennsylvania*

| - Amyotrophic lateral sclerosis - Anxiety disorders - Autism - Cancer, including remission therapy - Crohn’s disease - Damage to the nervous tissue of the central nervous system (brain-spinal cord) with objective neurological indication of intractable spasticity, and other associated neuropathies - Dyskinetic and spastic movement disorders - Epilepsy - Glaucoma - HIV / AIDS - Huntington’s disease - Inflammatory bowel disease - Intractable seizures - Multiple sclerosis - Neurodegenerative diseases - Neuropathies - Opioid use disorder for which conventional therapeutic interventions are contraindicated or ineffective, or for which adjunctive therapy is indicated in combination with primary therapeutic interventions - Parkinson’s disease - Post-traumatic stress disorder - Severe chronic or intractable pain of neuropathic origin or severe chronic or intractable pain - Sickle cell anemia - Terminal illness - Tourette syndrome |
| --- |

* https://www.pa.gov/guides/pennsylvania-medical-marijuana-program/
